# Circadian Clock Genes Modulate Human Bone Marrow Mesenchymal Stem Cell Differentiation, Migration and Cell Cycle

**DOI:** 10.1371/journal.pone.0146674

**Published:** 2016-01-07

**Authors:** Helene Boucher, Valerie Vanneaux, Thomas Domet, Alexandre Parouchev, Jerome Larghero

**Affiliations:** 1 Assistance Publique-Hôpitaux de Paris, Hôpital Saint-Louis, Cell Therapy Unit, Paris, France; 2 University Paris Descartes, School of Pharmacy, Paris, France; 3 INSERM, CIC de Biothérapies (CBT-501) and UMR1160, Institut Universitaire d’Hématologie, Hôpital Saint-Louis, Paris, France; 4 University Paris Diderot, Sorbonne Paris Cité, Paris, France; University of Glasgow, UNITED KINGDOM

## Abstract

Many of the components that regulate the circadian clock have been identified in organisms and humans. The influence of circadian rhythm (CR) on the regulation of stem cells biology began to be evaluated. However, little is known on the role of CR on human mesenchymal stem cell (hMSCs) properties. The objective of this study was to investigate the influence of CR on the differentiation capacities of bone marrow hMSCs, as well as the regulation of cell cycle and migration capabilities. To that, we used both a chemical approach with a GSK-3β specific inhibitor (2’E,3’Z-6-bromoindirubin-3’-oxime, BIO) and a knockdown of *CLOCK* and *PER2*, two of the main genes involved in CR regulation. In these experimental conditions, a dramatic inhibition of adipocyte differentiation was observed, while osteoblastic differentiation capacities were not modified. In addition, cell migration was decreased in PER2^-/-^ cells. Lastly, downregulation of circadian clock genes induced a modification of the hMSCs cell cycle phase distribution, which was shown to be related to a change of the cyclin expression profile. Taken together, these data showed that CR plays a role in the regulation of hMSCs differentiation and division, and likely represent key factor in maintaining hMSCs properties.

## Introduction

Circadian rhythms (CR) allow organizations to adapt to the periodic nature of the environment and anticipate the external light-dark cycle [1]. These endogenous lilting allow the regulation of physiological processes such as glucose homeostasis [2] and cell cycle [3]. The circadian clock is intimately involved in the control of metabolic and physiological process and disruption can be either the cause or the effect of various disorders including metabolic syndrome [4] or cancer predisposition [5]. The circadian system in mammals is a complex hierarchical system organized around a set of neurons coupled at the suprachiasmatic nuclei (SCN) responsive to stimuli through the retinohypthalamic tract. However, in addition to the SCN, independent oscillators have been shown to operate in peripheral tissues [6,7] and at the cellular level [8–10].

The mammalian circadian clock consists in a complex transcriptional, post-transcriptional, and translational auto-regulatory feedback loop [11]. The first level of control is organized by transcription factors belonging to the superfamily of nuclear receptors, including the retinoic orphan receptor-related receptors (RoRs), activator of the loop factor, and REV-ERB which act as a repressor of the feedback control loop [12] and is activated by the GSK-3β protein through phosphorylation. These factors targets BMAL1, which dimerization with CLOCK activated transcription of the *Per* and *Cry* genes through E -box element [13,14]. The PERs (Period) and CRYs (Cryptochrome) proteins then inhibits CLOCK:BMAL1 dimer after oligomerization, phosphorylation and nucleus translocation [15,16]. The turnover of PERs and CRYs inhibitory proteins provides a cyclicality of elements that participates in sustaining the 24 hours rhythm in mammals [1].

Several studies have suggested a link between CR and physiological processes, and demonstrated that genes controlling metabolic processes displayed CR, particularly those involved in lipid biosynthesis and metabolism [17]. Indeed, invalidation of *Clock* gene in rodents lead to metabolic syndrome such as dyslipidemia [18]. A loss of the rhythmicity of the PPARα transcription factor expression, involved in the regulation of adipocyte function, has also been reported [19]. In humans, an association between C*LOCK* gene polymorphism and predisposition to obesity [20], and between PER2 polymorphism and abdominal obesity [21] have been described.

CR has also been shown to regulate osteogenic potential. Inhibiting *Bmal1* promotes osteocytic differentiation [22], and *Per1*^*-/-*^*Bmal1*^*-/-*^ mice showed a significant increase of bone volume related to an increase of osteoblast progenitors proliferation [23].

These studies highlighted the role of clock genes in the regulation of cell proliferation and division, through the control of most of cyclin, CDKs and tumor suppressor genes, which displayed circadian rhythmicity [3]. Indeed, *Cry*^-/-^ rodents are characterized by a decreased cell proliferation and division, which lead to an increased time for liver regeneration compared to wild-type animals [24]. On a more specific stem cell point of view, a link has been reported between circadian rhythmicity and bone marrow hematopoietic stem cells (HSCs) output and homing [25]. CR gene expression was also found in HSCs, bone marrow or adipose-derived MSCs, embryonic stem cells and cancer stem cells [26]. However, the mechanisms by which CR may regulate stem cell proliferation, division, renewal, and differentiation properties, remain to be elucidated.

The objective of our study was to investigate whether CR control human hMSCs differentiation capacities, regulate cell cycle, and play a role in cell migration abilities.

## Material and Methods

### Mesenchymal stem cells culture and reagents

In our study, primary bone marrow mesenchymal stems cells (MSCs) were used in all experiments. The study was approved by Saint-Louis Hospital scientific committee. Bone marrow (BM) samples were harvested from washed filters used during BM graft processing for allogeneic transplantation after healthy donor written informed consent according to approved institutional guidelines (Assistance Publique—Hôpitaux de Paris, Paris, France, and French Ministry of Research number DC2009-929). For human mesenchymal stem cells (hMSCs) isolation, BM cells obtained after Ficoll (EuroBio) were cultured at an initial density of 5.10^4^ cells/cm^2^ in complete culture medium: α-Minimum Essential Medium (α-MEM) (Gibco) with 10% Fetal Bovine Serum (FBS) (Hyclone), 1 ng/ml basic Fibroblast Growth Factor (bFGF) (R&D System) and 1% Antibiotic-Antimycotic solution (ATB-ATM solution) (Gibco) (αMEMc medium). After 24–48 h, non-adherent cells were removed and the medium was changed. Cultures were fed every 2 or 3 days until 80% confluence. Adherent cells were then trypsinized (HyClone) harvested and cultured by seeding 5.10^3^ cells/cm^2^. Cells were expanded for 1 to 5 passages in 37°C with 5% CO_2_ culture conditions.

2’E,3’Z-6-bromoindirubin-3’-oxime (BIO) (Sigma-Aldrich), a selective inhibitor of the GSK-3β protein, was used at concentrations of 2 μM and 1 μM for adipogenic and osteogenic differentiation assays, respectively.

### Cell synchronization

hMSCs were cultured until 70–80% confluence. Culture medium was replaced with FBS free medium and cells were incubated overnight. For clock synchronization, cultures were rinsed with Phosphate-Buffered Solution (PBS) (Eurobio) and changed to 50% FBS medium for 1h incubation. After two PBS washing, cells were placed in fresh α-MEMc medium. Samples were taken every 6h for 36 h after serum shock. Serum shock was considered as time 0 (T0). Harvested cell samples were immediately frozen at -80°C and kept until RNA isolation.

### In vitro differentiation

#### Adipogenic differentiation

hMSCs were incubated in 6-well microplates in DMEM low glucose (Gibco) with 20% FBS, 60 μM indomethacin (Sigma-Aldrich), 0,5 mM isobuthyl methylxanthine (IBMX) (Sigma-Aldrich), 10^−6^ M dexamethasone (Mylan) and 1% ATB-ATM solution. Medium was changed every two days. At day 21, cells were stained or frozen at -80°C until RNA isolation. For staining, cells were fixed in 4% paraformaldehyde (PAF) (Electron Microscopy Sciences) and stained in oil red O solution (Bio-Optica).

#### Osteogenic differentiation

To induce osteogenic differentiation, hMSCs were incubated in 6-well microplates in DMEM low glucose with 10% FBS, 50 μg/mL ascorbic acid (Sigma-Aldrich), 10^−7^ M dexamethasone, 1% ATB-ATM solution and 3 mM inorganic phosphate (Sigma Aldrich). Medium was changed every two days. At day 21, cells were stained or frozen at -80°C until RNA isolation. For staining, cells were fixed in 4% PAF and stained in alizarin red solution (Sigma Aldrich) at pH 4.2.

### Gene expression assay

#### RNA Isolation and reverse transcription

RNA isolation was performed by using RNeasy Mini Kit (Qiagen). RNA concentration was determined spectrophotometrically at 260 nm and purity using the 260/280 nm ratio (Nanodrop, Thermo Scientific). Total RNA (500 ng) was reverse transcribed using TaqMan gene Expression Assays kit (Applied Biosystem).

#### Real-Time PCR

Real-time quantitative PCR analyses were performed on a TaqMan (Applied Biosystem). PCR reagents were TaqMan Universal PCR Master Mix No Amperase UNG and TaqMan Gene Expression Assay-On-Demand^™^ (Applied Biosystem). The initial step of PCR was a 10min hold at 95°C followed by 40 cycles of PCR amplification. PCR reactions were performed in triplicate in 96-wells plates. Analyses were performed using standard curves obtained from hMSCs cDNA with *Hmbs* (Hs00609297_m1) as the normalizing endogenous control. Fold change relative was calculated based on the 2^(–ΔΔCt)^ method. Pre-designed TaqMan gene expression assays from Applied Biosystems were: CR: *Clock* (Hs00231857_m1), *Bmal1* (Hs00154147_m1), *Per1* (Hs00242988_m1), *Per2* (Hs00256143_m1), *GSK-3β* (Hs01047719_m1); Osteogenic differentiation: *Alkaline Phosphatase* (Hs01029144_m1), *Osteocalcin* (Hs00609452_g1), *Runx2* (Hs00231692_m1); Adipogenic differentiation: *Fabp4* (Hs01086177_m1), *Pparγ* (Hs01115513_m1), *C/Ebpα* (Hs00269972_s1) and *Gapdh* (Hs99999905_m1).

### Flow Cytometry

Cells were detached with trypsin, fixed with 4% PAF for 10min and then washed twice with PBS. Cells were re-suspended in PBS with 0.5% FBS. Cells were labeled with the following anti-human antibodies: CD105-APC, CD73-APC, CD90-APC, CD44-APC, CD34-APC, and CD31-APC (Miltenyi), CD45-APC (Becton Dickinson) for immunophenotyping assays; CD49a-APC and CD49d-APC (Miltenyi), CD106-APC and CD54-APC (Becton Dickinson) for adherence assays; Rabbit anti-p21, Mouse anti-p27, Mouse anti-Cyclin B1, Rabbit anti-Cyclin D1 (all from Cell Signaling), and Rabbit anti-p19 (Upstate) for cell cycle assays. Donkey anti-Mouse IgG DyLight650 and Donkey anti-Rabbit IgG DyLight650 (1:200 dilution for each, Thermo Scientific) were used as secondary antibodies when needed. Isotype antibodies served as respective controls. For intracellular labeling, cells were permeabilized with PBS/0.1% Triton X100 solution (BioRad). Cells were acquired on a FACS Scan flow cytometry analyzer (FACs Calibur, Becton Dickinson) and analyzed using CellQuestPro software (Becton Dickinson).

### Immunofluorescence experiments

hMSCs were fixed in 4% PAF for 10min, permeabilized and blocked in 0.1% Triton X100, 5% FBS solution for 30min, washed twice with PBS, incubated with primary antibodies overnight at 4°C, and then incubated with secondary antibodies for 1h at room temperature. Cells were washed 3 times with PBS and mounted on cover slips with mounting medium Glycergel (Dako) and DAPI (Roche).

The following antibodies were used: Goat anti-CLOCK, Goat anti-BMAL1, Goat anti-PER1, Mouse anti-PER2 (1:50 dilution for each, all purchased from Santa Cruz Biotechnology), combined with appropriate secondary antibodies: donkey anti-goat FITC, donkey anti-goat Cy3 and donkey anti-mousse FITC (1:100 dilution for each, all purchased from Thermo Scientific).

### Lentiviral transduction

Cells were plated in 24-wells plate at 15.10^3^ cells/cm². hMSCs were incubated with lentiviral particles for 8h or 12h according to manufacturer’s protocol. Transduction efficiency was determined by the percentage of GFP+ cells using flow cytometry. Twenty-four hours after infection, 5 μg/mL puromycin (Life Technologies) was added for cell selection. Stable cell lines were obtained after 2 weeks. The following particles were used: VGM5524-Mouse GIPZ viral particles (Clock), VGH5523-Human GIPZ viral particles (Per2), Non-silencing GIPZ Lentiviral shRNA Negative Control (viral particles) (RHS4348), GAPDH GIPZ Lentiviral shRNA Positive Control (viral particles) (RHS4372) (all purchased from Thermo Scientific).

### Cell cycle

hMSCs were harvested, resuspended in 2 mL cold 70% ethanol and stored at –20°C until analysis. Before analysis, cells were washed and incubated in PBS containing Propidium Iodide (100 μg/mL) (Invitrogen) and RNase A (100 μg/mL) (Roche). A FACS Calibur Cytometer operated with CellQuestPro software was used for data collection.

### Migration studies

#### Wound healing

hMSCs where plated at 5.10^3^ cells/cm² in 24-wells plates in α-MEMc medium. At 100% confluence, a linear wound was made by scratching. Cells were washed twice with PBS and incubated with fresh medium for 24h. Cells were then visualized by microscopy and healing area was measured.

#### Transwell assay

hMSCs were incubated in αMEM-medium for 4h, trypsinized and re-suspended in α-MEM medium. 5.10^4^ cells were plated in the upper chamber which was placed in a 24-wells culture dish containing αMEMc medium in the presence or absence of 100 ng/mL Stromal Cell-derived Factor-1 (SDF-1). After staining with DAPI, invasive cells count was performed at 24h by fluorescence microscopy (10x magnification).

### Statistical Analysis

Data are expressed as the mean ± standard derivation. Statistical analyses were performed using the Student’s *t*-test, Wilcoxon-Mann Whitney test and one-way analysis of variance (ANOVA) at confidence level of 95% (R software). P values ≤ 0.05 were considered statistically significant.

## Results

### hMSCs characterization

**Fig 1:**
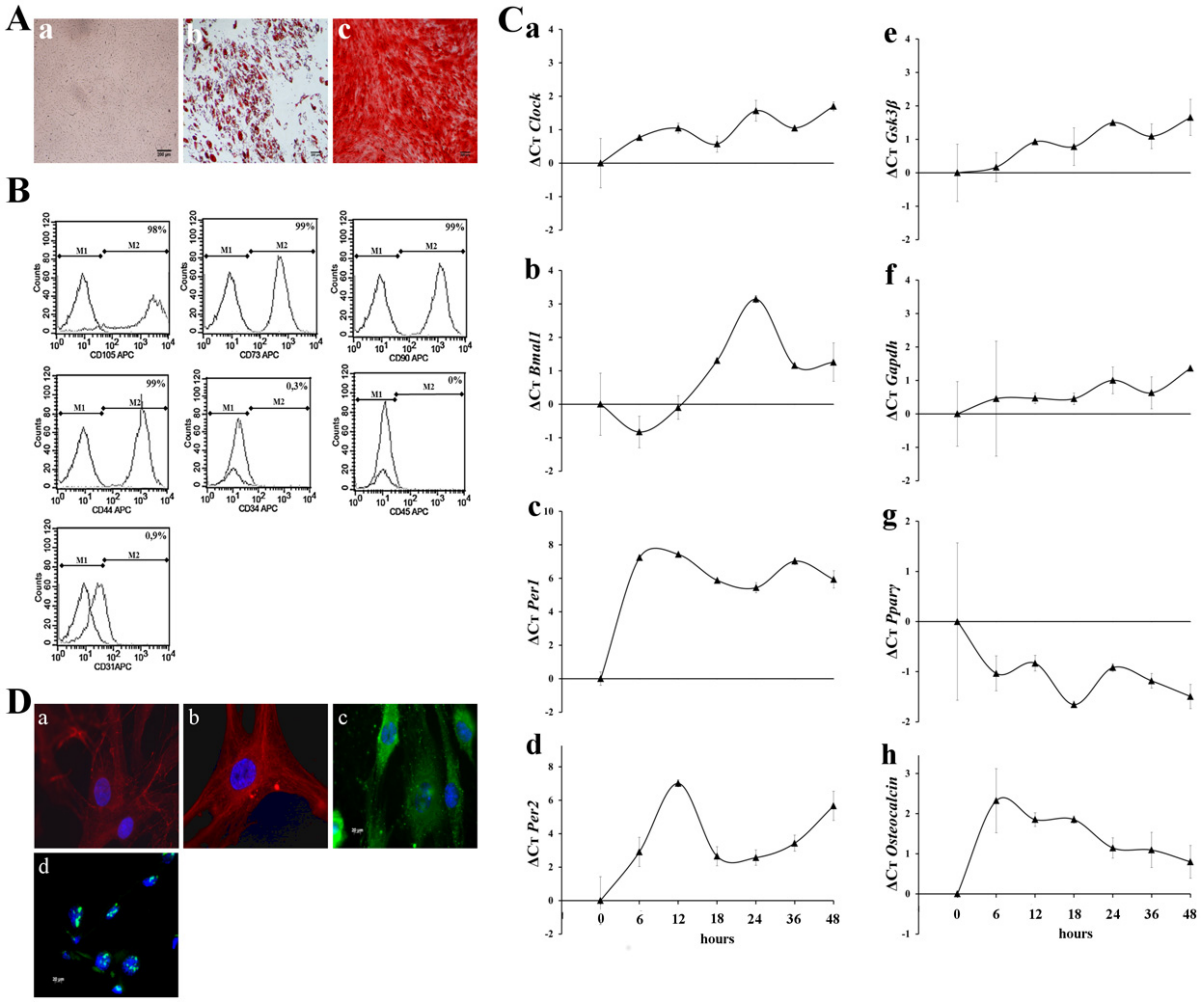
hMSCs characterization and circadian rhythms genes and protein expression. (A) hMSCs differentiation capacities. (a) hMSCs morphology in standard culture conditions. (b) Day 21 adipogenic differentiation, assessed by oil red O staining. (c) Osteogenic differentiation after 21 days, evaluated by alizarin red staining. Scale bar = 200 μm (B) hMSCs antigen expression characterization CD105, CD73, CD90, CD44, CD34, CD45 and CD31 by flow cytometry. The percentage of markers expression is given compared to isotype control. (C) Quantitative RT-PCR showing temporal expression profiles of clock-associated genes -*CLOCK* (a), *BMAL1* (b), *PER1* (c), *PER2* (d), *GSK-3β* (e)- and *GAPDH* (f), *PPARγ* (g) and *OSTEOCALCIN* (h) genes. Data, obtained during a 36 hours period, are expressed as the mean of ΔCt ± SEM calculated from the Ct value of each gene at T0 (serum shock). Bars represent means of 3 independent experiments. (D) Immunofluorescence analysis of circadian proteins -CLOCK (a), BMAL1 (b), PER1 (c) and only PER2 (d)- in hMSCs, 36 hours after starvation. Cell nuclei were visualized by DAPI (blue), circadian protein by FITC (green) or Cy3 staining (red). Scale bar = 20 μm.

Isolated hMSCs displayed adhesion capabilities to plastic and showed the typical fibroblast-like cell morphology (Fig 1A). hMSCs differentiation capacities along the adipogenic and osteogenic lineages was confirmed by their ability to exhibit lipid droplets stained with oil red O and calcium mineralization after staining with alizarin red solution, respectively (Fig 1A). As expected, hMSCs expressed the membrane antigens CD105 (98% ± 0.1%), CD73 (99% ± 0.2%), CD90 (99% ± 0.01%), and CD44 (99% ± 0.05%) and were found negative for the expression of hematopoietic and endothelial markers CD34 (0.3% ± 0.03%), CD45 (0% ± 0.02%) and CD31 (0.9% ± 0.9%) (Fig 1B), which is consistent with the criteria proposed by ISCT for MSCs characterization [27].

### Expression of circadian genes and protein in hMSCs

The relative expression of *CLOCK*, *BMAL1*, *PER1*, *PER2*, *PPARγ*, *OSTEOCALCINE* and *GSK-3β* genes and CLOCK, BMAL1, PER1 and PER2 proteins was studied in individual cultures of BM hMSCs (n = 3). Gene expression was evaluated by qRT-PCR in cells synchronized by serum shock (T0) and then followed every 6 hours during 36 hours under starvation. Results showed a rhythmic expression patterns for *CLOCK*, *BMAL1*, *PER1* and *PER2* genes (Fig 1B). Rhythmicity was observed for all clock genes, with a period of oscillation (P) of 24 hours for *CLOCK*, *BMAL1* and *PER1*, and 18 hours for *PER2*. Dimerization partners *CLOCK* and *BMAL1* were shown to have a phasic expression. Importantly, *CLOCK* and *BMAL1* transcripts were seen to be antiphasic to their repressors *PER1* and *PER2*. These results indicate that expression and rhythmicity of clock gene arise at the cellular level in hMSCs. Similar results were obtained for *GSK-3β*, but not for differentiation gene *PPARγ* and *OSTEOCALCIN* and *GAPDH* for which circadian oscillation were not observed.

Immunofluorescence staining confirmed the clock proteins expression in hMSCs. CLOCK and BMAL1 were observed to be located primarily in the cytoplasm. PER1 was detected in both the cytoplasm and the nucleus. PER2 was observed to be located only in the nucleus and particularly in the nucleoli (Fig 1C).

### Impact of BIO on adipogenic and osteogenic differentiation

**Fig 2:**
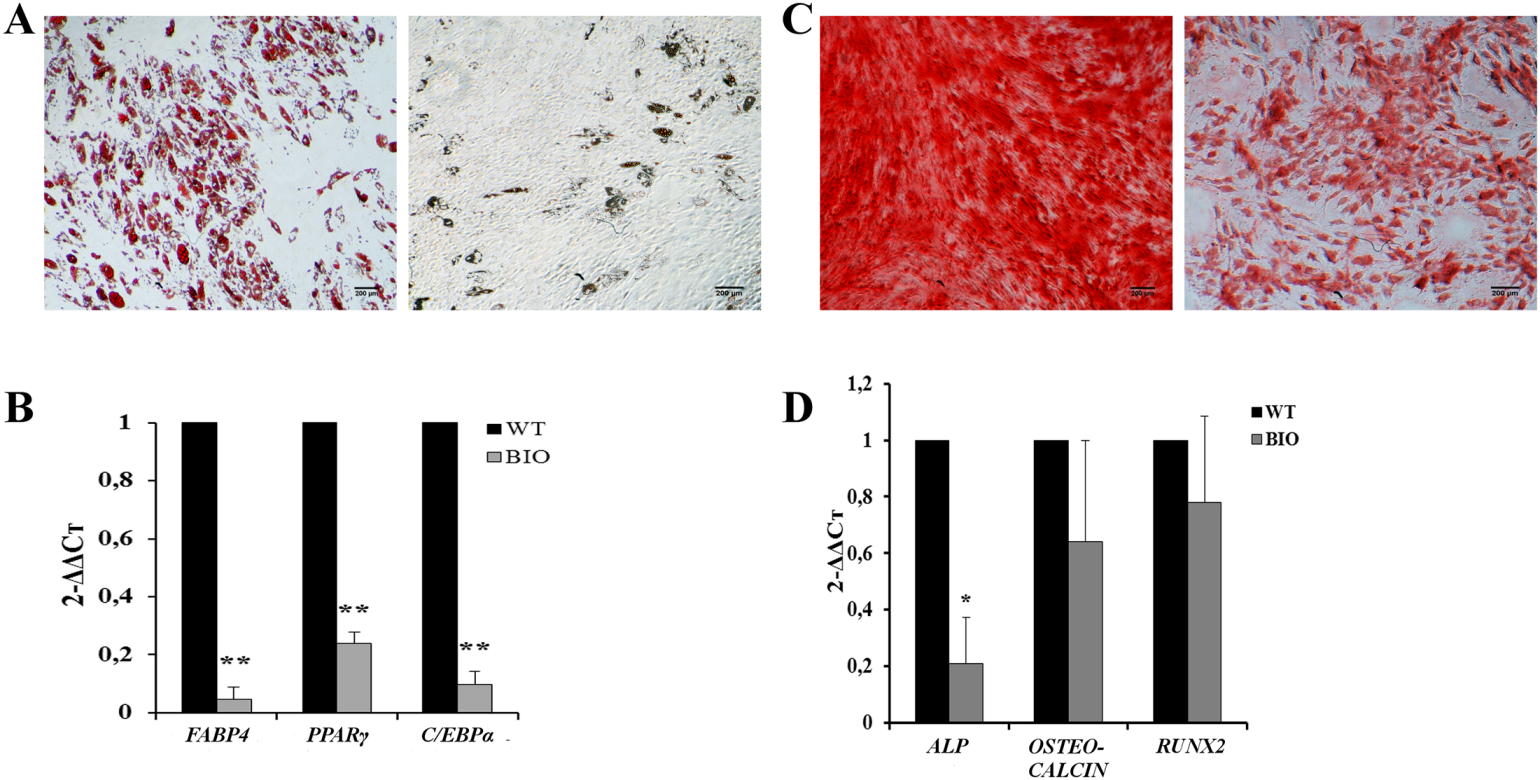
Inhibition of GSK-3β induces a blockade of hMSCs-adipogenic differentiation and attenuates osteogenic differentiation. (A) Adipogenic differentiation of hMSCs cultured in the absence (a) or in the presence of 2 μM BIO (b). Cells were stained with oil red O. Photographs are representative of three independent experiments. Scale bar = 200 μm (B) Expression of key adipogenic transcription factors in cells treated or not with 2 μM BIO. RT-qPCR data are expressed as the mean of 2^(–ΔΔCt)^ ± SEM. Endogenous gene: *HMBS*. Bars represent means of 3 independent experiments. **: p<0.01. (C) hMSCs osteogenic differentiation at days 21. Cells were cultured in standard condition (a) or in the presence of 1 μM BIO (b) and stained with alizarin red. Photographs are representative of three independent experiments. Scale bar = 200 μm (D) Expression of key osteogenic transcription factors in cells treated or not with 1 μM BIO. RT-qPCR data are expressed as the mean of 2^(–ΔΔCt)^ ± SEM. Endogenous gene: *HMBS*. Bars represent means of 3 independent experiments. *: p<0.05.

In an attempt to modulate the CR through β-catenin signaling, hMSCs were treated with BIO, a specific GSK-3β inhibitor that acts by mimicking Wnt signaling. After 21 days of adipogenic differentiation induction in the presence of 2 μM BIO, a dramatic reduction of hMSCs differentiation was observed compared to untreated cells (Fig 2A). At the transcriptional level, the inhibition of differentiation along the adipogenic lineage was associated with a significant decrease of *FABP4*, *PPARγ* and *C/EBPα* (p<0.01), three genes known to have a key role in adipocytic maturation (Fig 2B).

Similar experiments were performed in order to induce hMSCs osteogenic differentiation. After 21 days of osteogenic differentiation in the presence or absence of 1 μM BIO, cells were stained with alizarin red. Calcium mineralization was slightly decreased in treated cells, as compared to control (Fig 2C). To confirm these results at the molecular level, A*LKALINE PHOSPHATASE* (*ALP*), *OSTEOCALCINE* and *RUNX2* gene expression were evaluated. As shown in Fig 2D, only *ALP* expression was significantly diminished when compared to untreated cells. Because *ALP* is a late marker of osteocyte differentiation, these results suggest that BIO likely inhibits the final osteoblasts maturation rather than the early steps of this process.

### Knockdown of circadian genes and hMSCs differentiation capacities

**Fig 3:**
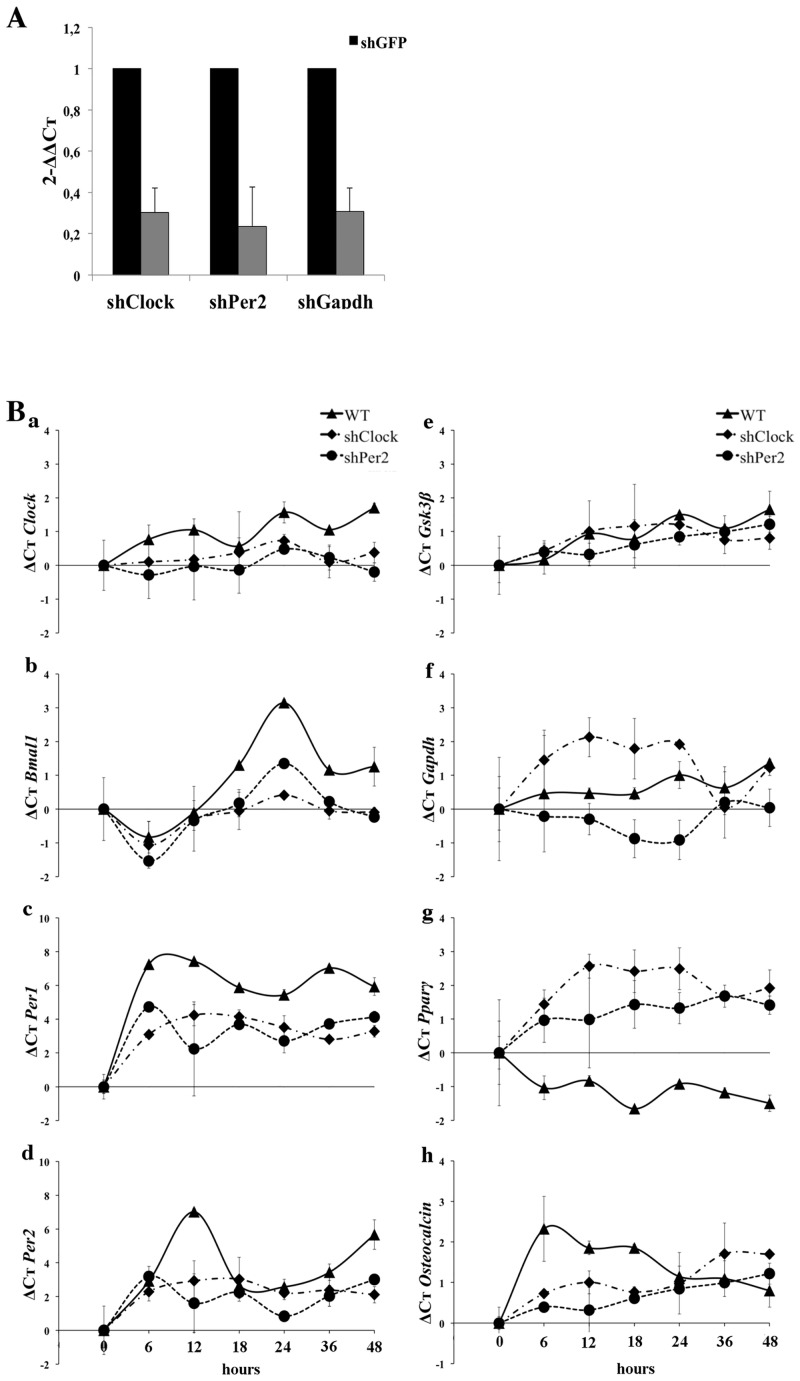
Gene expression in knockdown hMSCs. (A) *CLOCK*, *PER2* and *GAPDH* mRNA levels in hMSCs, normalized to shGFP. Data are expressed as the mean of 2^(–ΔΔCt)^ ± SEM. Endogenous gene: *HMBS*. Bars represent means of 3 independent experiments. *: p<0.05. (B) Expression profiles of clock-associated genes -*CLOCK* (a), *BMAL1* (b), *PER1* (c), *PER2* (d), *GSK-3β* (e)- and *GAPDH* (f), *PPARγ* (g) and *OSTEOCALCIN* (h) in WT, shClock and shPer2 hMSCs (T0 = serum shock). Data are expressed as the mean of ΔCt ± SEM calculated from the Ct value of each gene T0. Bars represent means of 2 independent experiments.

In order to specifically analyze the potential role of different regulatory CR genes on hMSCs differentiation, knockdown of *CLOCK* and *PER2* (shClock and shPer2 respectively) were performed by using a shRNA strategy. shGFP and shGapdh were used as control. A mean knockdown efficiency of 70% was obtained for the targeted genes (Fig 3A).

Expression of *CLOCK*, *BMAL1*, *PER1*, *PER2*, *GSK-3β*, *PPARγ* and *OSTEOCALCIN* gene was then evaluated in synchronized shClock and shPer2 hMSCs and compared to those obtained for WT cells. As expected, *CLOCK* expression was downregulated in shClock hMSCs (Fig 3Ba). *CLOCK* knockdown induced a downregulation of *BMAL1*, the dimerization partner of *CLOCK*, and a decrease of the oscillation amplitude, without modification of the period oscillation (Fig 3Bb). The antiphasic oscillations of repressor of *CLOCK*, *PER1* and *PER2*, was not modified in shClock hMSCs. However, a lengthening of the *PER1* and *PER2* period of oscillation was observed compared to WT cells (P = 30 hours *versus* 24 hours for *PER1* and P = 30 hours *versus* 18 hours for *PER2*, respectively) (Fig 3Bc and 3Bd). This was associated with a decrease of the oscillation amplitude. Similar results were obtained for *GSK-3β* (Fig 3Be). The inhibition of *CLOCK* in hMSCs slightly modified *OSTEOCALCIN* expression but was associated with a significant overexpression of *PPARγ*, suggesting a regulatory role of *CLOCK* on this key player of adipocyte differentiation (Fig 3Bg and 3Bh).

When using shPer2 hMSCs, a decrease of the *PER1* oscillation period was observed, from 24 hours to 12 hours (Fig 3Bc). *CLOCK* and *BMAL1* expression profile was not significantly modified (Fig 3Ba and 3Bb). shPer2 was shown to substantially impact *GSK-3β* expression, which oscillation disappeared (Fig 3Be). Lastly, *PER2* inhibition induced a similar profile of expression to that observed with shClock on differentiation genes, with an upregulation of *PPARγ* and a decreased of *OSTEOCALCIN* (Fig 3Bg and 3Bh).

#### Knockdown of CR genes inhibits adipogenic differentiation

**Fig 4:**
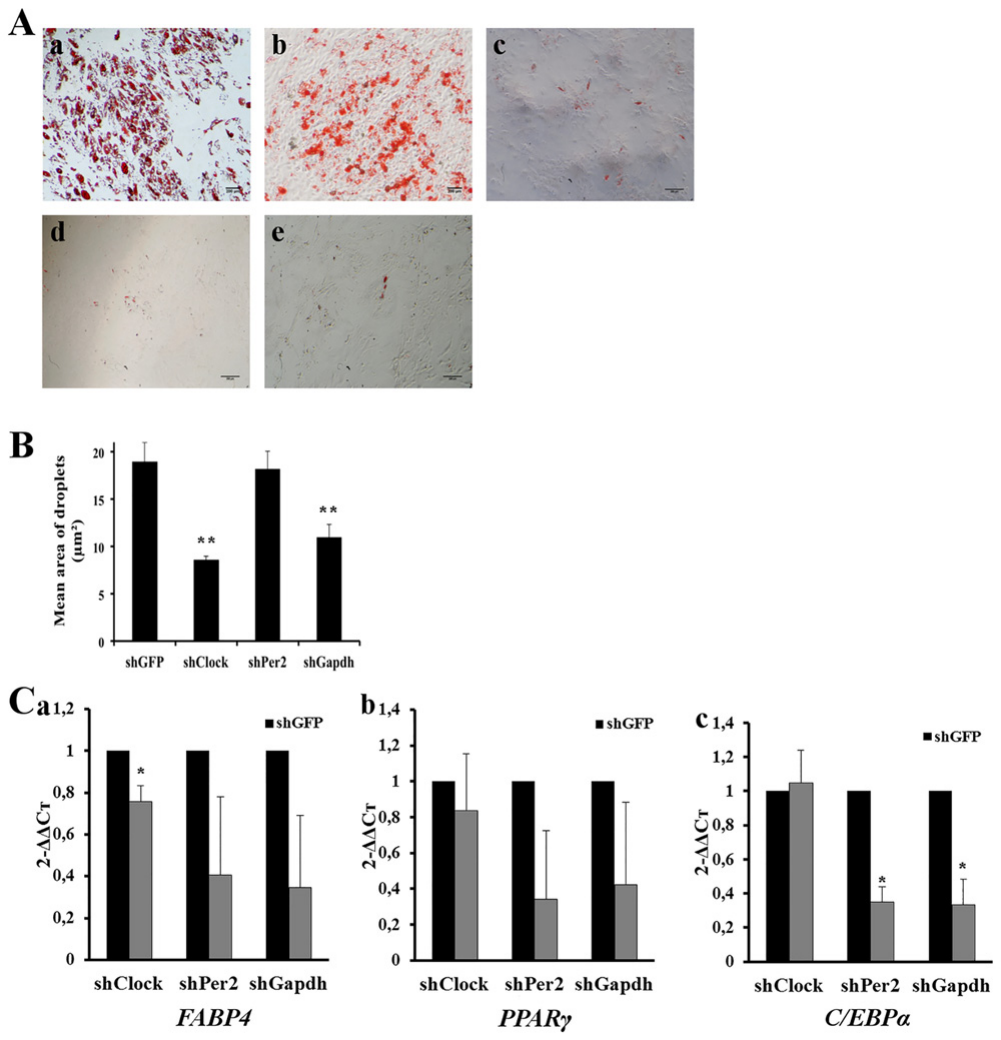
Circadian rhythm regulates adipogenic differentiation. (A) Influence of circadian genes inhibition on hMSCs adipogenic differentiation. WT (a), shGFP (b), shClock (c), shPer2 (d) and shGapdh (e) hMSCs were stained with oil red O after 21 days of differentiation. Scale bar = 200 μm. (B) Mean area (μm²) of each lipid droplets, determined with ImageJ^®^ software. (C) qPCR analysis of adipogenic differentiation markers: (a) *FABP4*, (b) *PPARγ* and (c) *C/EBPα*. Data are expressed as mean of 2^(–ΔΔCt)^ ± SEM and normalized to shGFP cells. Endogenous gene: *HMBS*. Bars represent means of 3 independent experiments. **: p<0.01, *: p<0.05.

WT, shGFP, shClock, shPer2 and shGapdh cells were engaged along the adipogenic differentiation. After 21 days of differentiation, WT and shGFP cells exhibited similar adipogenic differentiation capacities (Fig 4Aa and 4Ab). Conversely, the number of droplets observed in shClock, shPer2 and shGapdh cells was significantly decreased compared to WT and shGFP cells (Fig 4Ac–4Ae). In order to quantify the lipid droplets, a counting was performed by using ImageJ^®^ software and the mean area of each droplets per adipocyte was measured (Fig 4B). The mean area for shGFP cells was of 18.94 ± 2.06 μm^2^, similar to that of WT cells. Consistently to that was observed in Fig 4A, the droplet area was found significantly decreased in shClock and shGapdh cells compared to shGFP cells (8.54 ± 0.42 μm^2^ and 10.92 ± 1.42 μm^2^, respectively, compared to 18.94 ± 2.06 μm^2^, p<0.01). For shPer2, a decreased number of droplets was observed but the average droplets area per adipocyte was not modified compared to shGFP cells (18.17 ± 1.49 μm^2^ and 18.94 ± 2.06 μm^2^, respectively).

The role of gene knockdown was then evaluated on the expression of *FABP4*, *PPARγ* and *C/EBPα* adipogenic transcription factors. In shClock cells, a slight decrease, but not reaching statistical significance, was found for *FABP4* and *PPARγ* expression (Fig 4Ca and 4Cb), while no variation of expression could be found for *C/EBPα* compared to shGFP cells (Fig 4Cc). The decrease of *FABP4*, *PPARγ* and *C/EBPα* expression was more pronounced in shPer2 and shGapdh cells, consistently with results obtained with oil red staining (Fig 4Ca–4Cc). Taken together, these results confirm those obtained with BIO chemical inhibitor.

#### Knockdown of CR genes did not influence osteogenic differentiation

**Fig 5:**
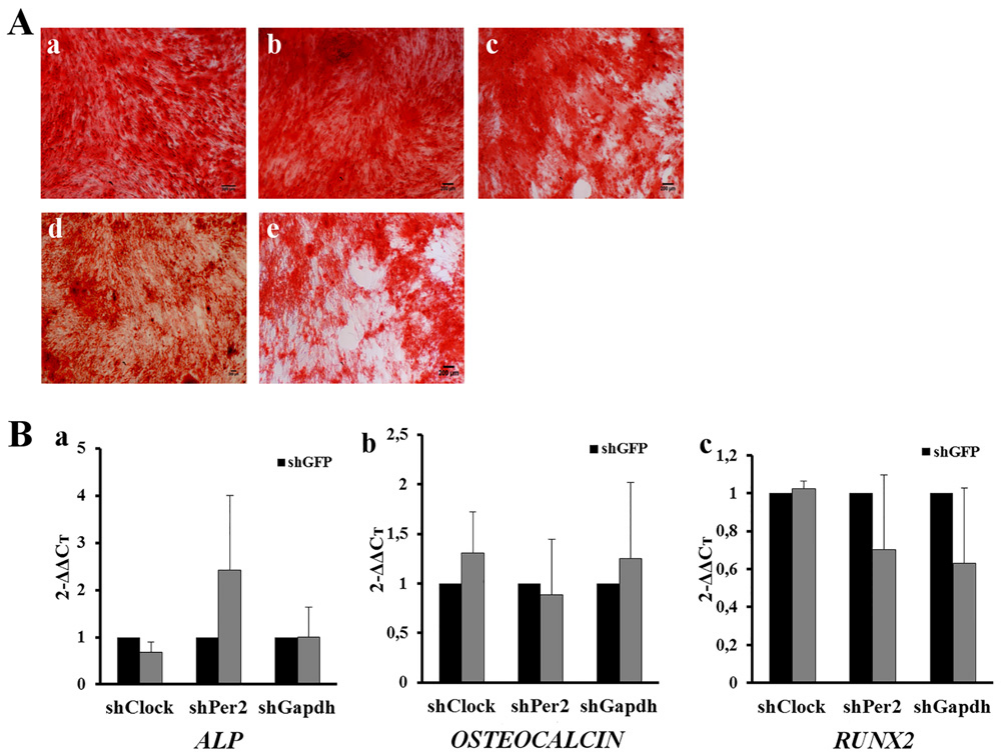
Role of circadian rhythm on osteogenic differentiation. (A) alizarin red staining after 21 days of osteogenic differentiation assessed on WT (a), shGFP (b), shClock (c), shPer2 (d), and shGapdh (e) hMSCs. Scale bar = 200 μm. (B) qPCR analysis of osteogenic differentiation markers: (a) *ALP*, (b) *OSTEOCALCIN* and (c) *RUNX2*. Data are expressed as mean of 2^(–ΔΔCt)^ ± SEM and normalized to shGFP cells. Endogenous gene: *HMBS*. Bars represent means of 3 independent experiments.

A similar experimental approach was used to evaluate hMSCs osteogenic differentiation capacities after gene knockdown. Qualitative alizarin red staining assay showed that hMSCs were equally capable to differenciate along the osteogenic lineages, whatever the shRNA used (Fig 5Aa–5Ae).

*ALP*, *OSTEOCALCIN* and *RUNX2* expression confirms these results. While some differences of expression were observed between shClock, shPer2, and shGapdh hMSCs compared to shGFP cells for some of the key osteogenic transcription factors, none were statistically different (Fig 5Ba–5Bc). These observations are in agreement with those obtained with inhibition of GSK-3β by BIO, and support evidence that influence of clock genes is likely not sufficient to substantially modify hMSCs osteogenic differentiation.

### Inhibition of CR genes modulated hMSCs adhesion and migration capacities

**Fig 6:**
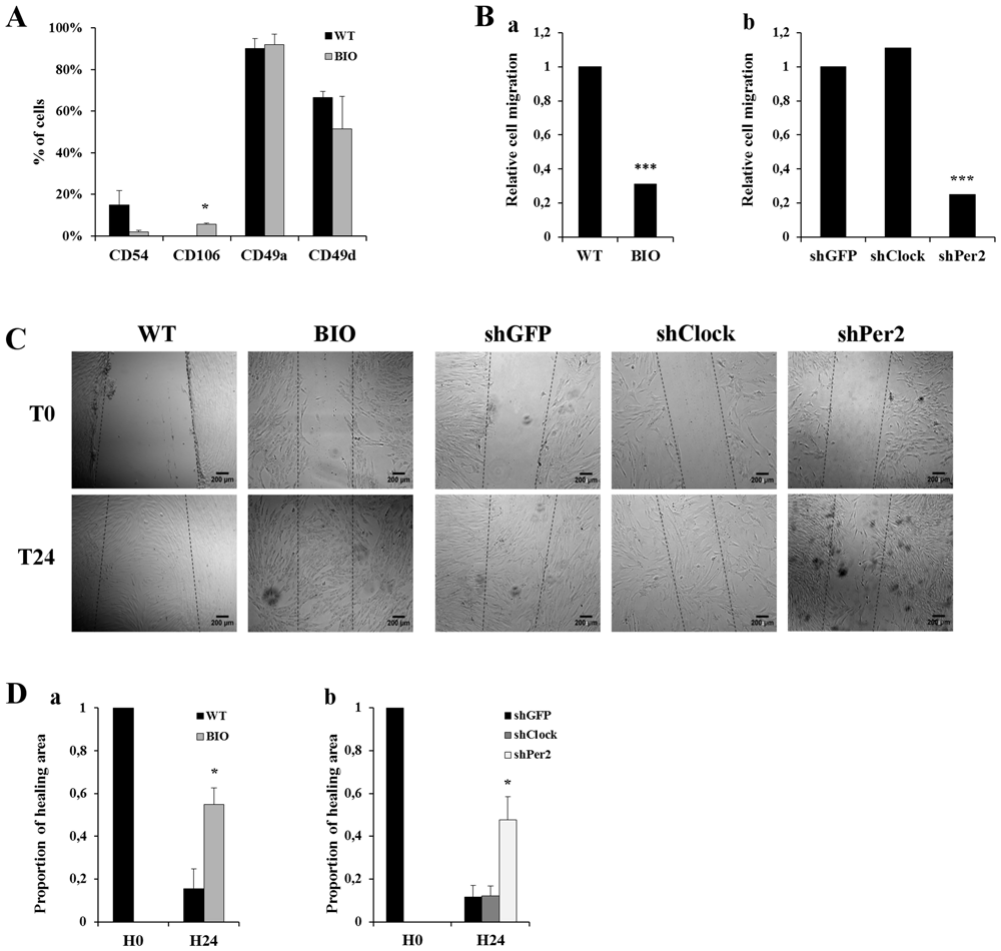
Analysis of circadian rhythm influence on cell adhesion and migration. (A) Expression of adhesion molecule CD54, CD106, CD49a and CD49d on hMSCs cultured without (WT) or in the presence of BIO. Bars represent means of 3 independent experiments. *: p<0.05. (B) Cell migration evaluated after 24 hours of stimulation with SDF-1. Graph represent the ratio of BIO-treated hMSCs migration compared to untreated cells (WT) (a) and to and that of shClock and shPer2 hMSCs compared to shGFP cells (b). Bars represent means of 3 independent experiments. ***: p<0.005. (C) Wound healing assay at T0 and T24 hours for WT, BIO-treated, shGFP, shClock and shPer2 hMSCs. Scale bar = 200 μm. (D) Proportion of healing area at T0 and at T24 hours for WT and BIO-treated hMSCs (a) and shGFP, shClock and shPer2 hMSCs (b). Bars represent means of 3 independent experiments. *: p<0.05.

hMSCs culture in the presence of BIO did not change cell morphology. However, a significant increase of the adhesion molecule CD106 expression was observed after BIO treatment compared to untreated cells (5.75% versus 0.1%, p <0.05), without modification of the other studied molecule (Fig 6A).

Interestingly, hMSCs migratory properties were modified both in culture with BIO and in shPer2 cells. Transwell analyses in the presence of SDF-1 showed a dramatic decrease of cell migration at 24 hours after BIO treatment compared to untreated cells (p<0.001) (Fig 6Ba) and in shPer2 hMSCs compared to shGFP cells (p<0.001) (Fig 6Bb). Contrarily, shClock did not significantly modified migration capacities (Fig 6Bb). To further investigate the effect of CR on cell migration, a wound-healing assay was performed. After 24 hours of culture, untreated cells as well as shClock and shGFP cells migrated and recovered the wound field, whereas BIO-treated cells and shPer2 cells only partially did (Fig 6C). Quantitative analysis of the wound area confirmed a significant difference between migration of the BIO-treated cells compared to controls (ratio H0/H24 = 0.20 ± 0.1 and 0.53 ± 0.07 for untreated and BIO-treated cells, respectively, p<0.05) (Fig 6Da). Similar results were obtained for shPer2 cells (ratio H0/H24 = 0.11 ± 0.05 and 0.47 ± 0.1 for shGFP and shPer2 cells, respectively, p<0.05) (Fig 6Db). Wound area was not found different between shGFP and shClock cells (ratio H0/H24 = 0.12 ± 0.04 in both condition).

### hMSCs cell cycle is regulated by CR genes

**Fig 7:**
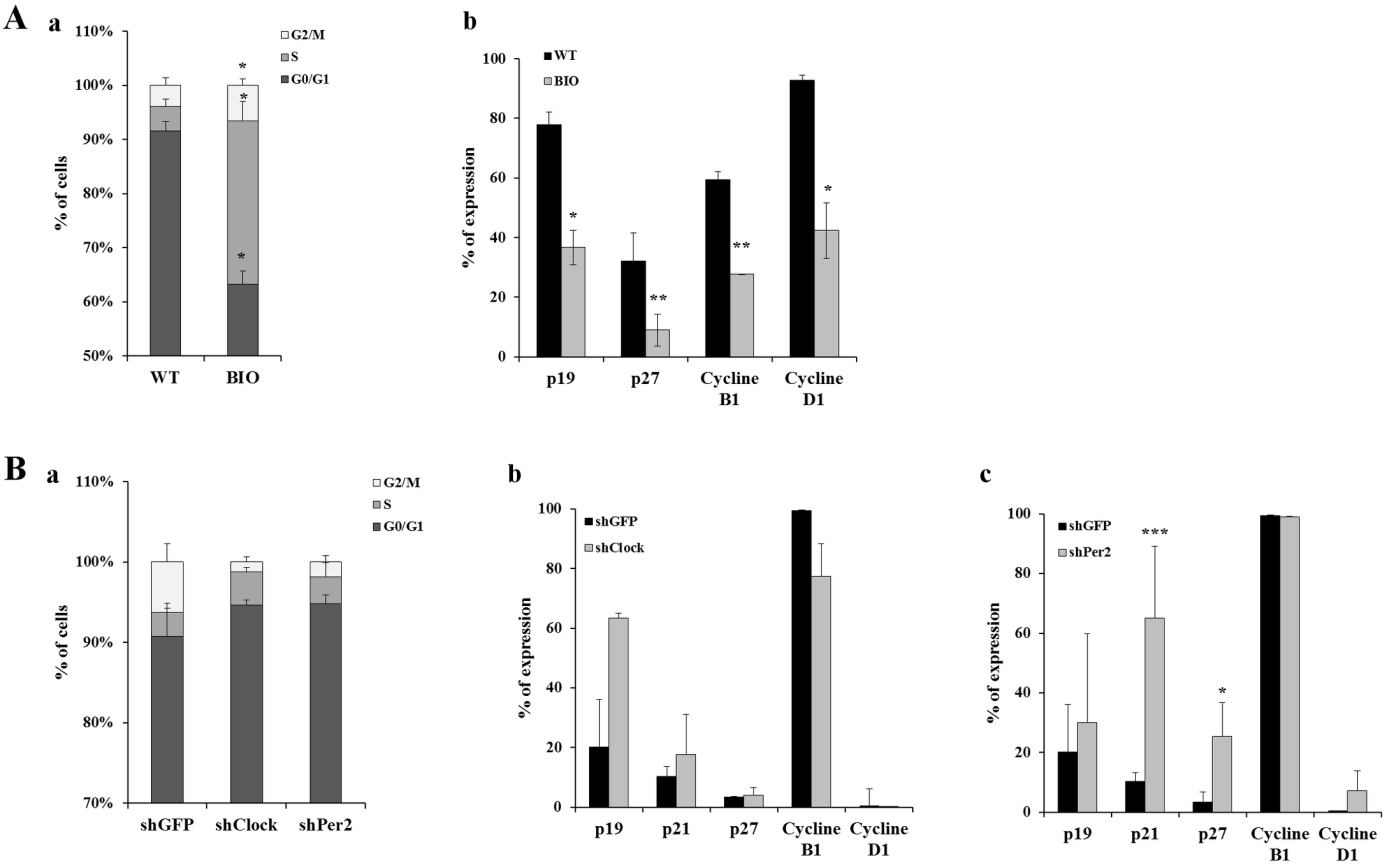
Effect of circadian rhythm gene modulation on hMSCs cell cycle. (A) Cell cycle studies and BIO treatment. (a) Histograms represent DNA contents according to the cell cycle phase G0/G1phase, S phase and G2/M phase for WT and BIO-treated hMSCs. Data are expressed as the mean of percentage ± SEM. Histograms represent means of 3 independent experiments. *: p<0.05. (b) Expression of cell cycle regulatory protein assessed by flow cytometry for WT and BIO-treated hMSCs. Data represent mean of 3 independent experiments. *: p<0.05; **: p<0.01. (B) Cell cycle studies and knockdown of circadian genes. (a) Histograms represent DNA contents according to the cell cycle phase G0/G1phase, S phase and G2/M phase for shGFP, shClock and shPer2 hMSCs. Data are expressed as the mean of percentage ± SEM. Histograms represent means of 3 independent experiments. (b-c) Expression of cell cycle regulatory protein assessed by flow cytometry for shGFP and shClock hMSCs (b) and shGFP and shPer2 hMSCs (c). Data represent mean of 3 independent experiments. *: p<0.05; ***: p<0.005.

The percentage of hMSCs in G0/G1, S, and G2/M phase of the cell cycle was evaluated after treatment with BIO or gene knockdown. We found that 91.5% ± 1.7% of control hMSCs were in G0/G1 phase, 4.5% ± 1.4% in S phase and 3.9% ± 1.4% in G2/M phase (Fig 7Aa). Twenty-four hours after BIO exposure, the percentage of cell in G0/G1 significantly decrease (63.9% ±2.4%, p<0.05). Conversely, the percentage of cell in S and G2/M phase significantly increase (30.5% ± 3.6% and 7.5% ± 0.2%, respectively, p<0.01 for both compared to control) (Fig 7Aa). These changes were associated with modifications of expression of proteins involved in cell cycle regulation. Indeed, p19, p27, CYCLIN B1 and CYCLIN D1 protein level significantly decreased in BIO-treated cells compared to control hMSCs (36.6% ± 5.7% versus 77.8% ± 4.2%, 8.9% ± 5.4% versus 32.1% ± 2.6%, 27.7% ± 0.12% versus 59.4% ± 1.7%, and 42.3% ± 9.33% versus 92.7% ± 1.9%, respectively, p<0.05) (Fig 7Ab). Similar results were obtained at the gene level (S1 Fig).

Interestingly, the profile of cell cycle phase in shClock and shPer2 cells was not significantly modified compared to shGFP cells (Fig 7Ba) or to untransduced hMSCs even if the number of cells in the GO/G1 phase was increased, which was correlated with a decrease of the S and G2/M phase. The fact that p19 level tend to be higher in shClock cells compared to shGFP (Fig 7Bb), and that a significant increase of p21 and p27 levels were observed in shPer2 cells compared to shGFP (Fig 7Bc), does not seem sufficient to induce a substantial modification of the cell cycle phase. No significant differences were observed for the expression of CYCLIN B1 and CYCLIN D1 between shClock, shPer2 and shGFP cells.

## Discussion

In this study, we demonstrated the presence of circadian oscillation of key genes of circadian loop in hMSCs. *In vitro*, serum-shock induced robust antiphase oscillations of *Clock/Bmal1* and *Per1/Per2* mRNA. In preliminary experiments on WT and shRNA hMSCs, we were able to show a constitutive expression of *CLOCK*, *BMAL1*, *PER1*, *PER2* genes at different time point without synchronization. However, in these conditions, gene expression was found to be too low and fluctuating for correctly evaluating gene oscillation. Therefore, we decided to use serum-shock synchronization in our experiments in order to study gene expression oscillation in a reproducible manner. Peripheral oscillations were already reported *in vitro*, particularly in murine fibroblasts, drosophila, murine and human stem cells [6,28–31]. We also showed in hMSCs that the activators of the CR loop, *CLOCK* and *BMAL1*, exhibited antiphasic mRNA expression compared to their repressors *PER1* and *PER2*, which is consistent with previous report [32–34].

We thus aimed to evaluate whether or not CR was involved in the regulation of hMSCs differentiation capacities. We showed that specific inhibition of GSK-3β by BIO dramatically downregulated *C/EBPα*, *FABP4* and *PPARγ*, known to act at different stages of adipocyte maturation [35]. This led to an inhibition of hMSCs differentiation along the adipogenic lineage, as revealed by the oil red O staining assay. These results are in agreement with previous reports showing that activation of Wnt pathway precludes cell differentiation into adipocytes [36–39]. The same approach was used to evaluate the role of GSK-3β inhibition on osteogenic differentiation. We found that BIO treatment reduced bone marrow hMSCs capacities to differentiate along the osteogenic lineage, partly through a downregulation of *ALP*. Our results are consistent with those obtained by Zaragosi et al. in human mesenchymal adipose derived stem cells [39]. Taken together, these results demonstrated that GSK-3β plays a central role in controlling differentiation potential and in maintaining undifferentiated hMSCs.

We then more precisely addressed the role of CR genes on hMSCs properties. In the context of lipid metabolism, it has indeed been shown that embryonic fibroblasts from *Bmal1*^*-/-*^ mice fail to differentiate into adipocytes [40], and that, both *in vitro* and *in vivo*, the lack of *Bmal1* results in reduced adipocytes differentiation and decreased lipid storage in adipocytes [41,42]. Interestingly, we find similar results in shClock hMSCs, the dimerization partner of *BMAL1*. A significant decrease in the number and the droplets area was observed, even if only *FABP4* expression was diminished. This apparent discrepancy may be explained by the fact that *CLOCK* can inhibit other genes involved in adipocyte differentiation and also act through its dimerization with *BMAL1*, which has not been inhibited in our study.

An inhibition of hMSCs adipocyte differentiation was also observed in shPer2 cells, with a decrease of the droplets number, but not of their size, associated with a downregulation of regulatory gene expression. *Per2* exerts its inhibitory function by blocking Pparγ recruitment to its target promoters and thereby its transcriptional activation. Altered lipid metabolism has been reported in *Per* deficient or mutant mice, but results still are contradictory and model dependent. Knockdown of *Per2* or *Per3* results in increased activation of adipogenic genes in adipose tissue [43,44], but a significant diminution expression of *Pparγ* in *Per1/2* null mice has also been reported [45]. *PER2* effect on adipogenic genes regulation thus appeared to be cell and tissue specific. Moreover, *Per2* is one of an actor in a complex network, and is thus dependent on the expression of other partners of the circadian loop. This has been exemplified, as an example, in *Clock* mutated mice in which an increased expression of *Per2* correlated with a decreased adipocyte differentiation [18,34].

The regulatory pathways involving CR genes along osteogenic differentiation are even less known in human cells. An increase of bone mass has been reported in *Per2*^*-/-*^ and *Cry2*^*-/-*^ mice [46], and clock genes were shown to mediate leptin-dependent bone formation [23]. In addition, mutations in the *Per1* and *2* genes result in uncontrolled *c-myc* signaling, overexpression of *G1 cyclins*, and increased osteoblast proliferation [47]. In our study, neither shPer2 nor shClock hMSCs displayed altered osteogenic differentiation, nor was modified gene expression of three main actors involved in this pathway. In all, our data clearly demonstrated that molecular clock regulates hMSCs adipogenic differentiation, but likely not osteogenic differentiation, at least in an *in vitro* culture model.

Studies in mammalian cells have shown interactions between the molecular regulation of cell cycle and the circadian clock. In our hMSCs model, we observed that cell culture in the presence of BIO induced a significant decrease of cells in G0/G1 phase and an increase of cells in S and G2/M phase. Similar results were obtained in murine embryonic fibroblasts [48], cancer cell lines [49], and immortalized pancreatic mesenchymal stem cells [50]. However, a chemical inhibition of GSK-3β cannot totally rely on the influence of the circadian clock on cell cycle regulation. For this reason, we performed the same analyses in shClock and shPer2 cells. We showed that inhibiting *CLOCK* or *PER2* influenced cell cycle regulation, perturbing G1/S and at G2/M transitions. This is consistent with *in vitro* and *in vivo* studies showing a deregulation of the G1/S transition after downregulation of *Per2* in murine breast cancer cells [51], and cyclin D1-dependent cell cycle deregulation in *Per2*^*-/-*^ mice models [52]. However, it has to be kept in mind that *in vitro* studies were obtained in most cases with murine cell lines, immortalized cell lines or human cancer cells. In our study, primary bone marrow MSCs were used in all experiments. This may explain discrepancies between some of our results and already published data.

We acknowledge that our study has however limitations, the most important being that not all main actors of molecular were knockdown. However, the physiological implications of our findings indicated the potential for pharmacological intervention with inhibitor of GSK-3β and specific target of circadian genes.

## Supporting Information

S1 Fig Effect of circadian rhythm gene modulation on hMSCs cell cycle gene expression. qPCR analysis of cell cycle gene regulator: (A) Cells treated with 2 μM BIO (B) shClock and shPer2 hMSCs. Data are expressed as mean of 2^(–ΔΔCt)^ ± SEM and normalized to WT cells or shGFP cells. Endogenous gene: *HMBS*. Bars represent means of 3 independent experiments. *: p<0.05; **: p<0.01.
(TIF)
