# Supplementary figures and images for: Circadian Clock Genes Modulate Human Bone Marrow Mesenchymal Stem Cell Differentiation, Migration and Cell Cycle
Source: PLoS One. 2016 Jan 7;11(1):e0146674. doi: 10.1371/journal.pone.0146674 (PMC4704833; doi:10.1371/journal.pone.0146674)

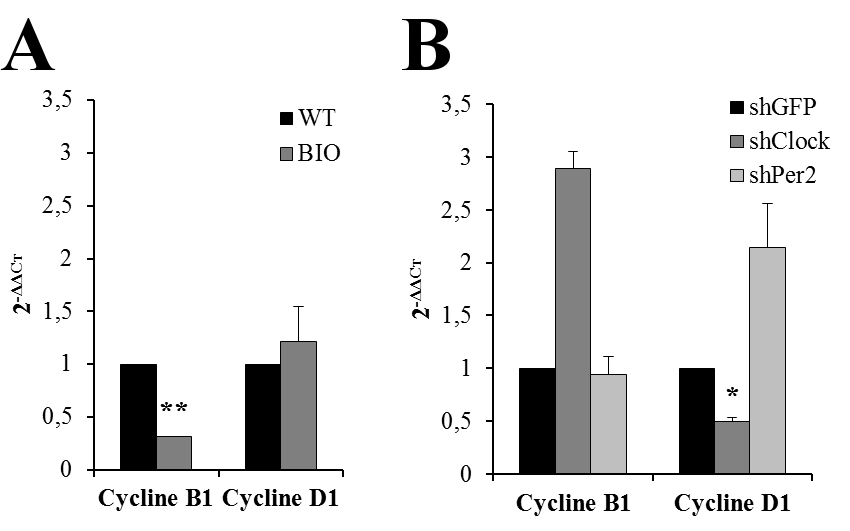

Supplement: S1 Fig — qPCR analysis of cell cycle gene regulator: (A) Cells treated with 2 μM BIO (B) shClock and shPer2 hMSCs. Data are expressed as mean of 2(–ΔΔCt) ± SEM and normalized to WT cells or shGFP cells. Endogenous gene: HMBS. Bars represent means of 3 independent experiments. *: p<0.05; **: p<0.01. (TIF) [file pone.0146674.s001.tif]
